# Positive Correlation Between Fecal Estrogen and Glucocorticoid Metabolites in a Female Clouded Leopard

**DOI:** 10.1002/zoo.21877

**Published:** 2024-11-08

**Authors:** Laura E. Shipp, Nicole P. Boisseau, Corinne P. Kozlowski, Dustin W. Shipp, Ashley D. Franklin, Jilian M. Fazio, Janine L. Brown

**Affiliations:** ^1^ Loveland Living Planet Aquarium Draper Utah USA; ^2^ Center for Species Survival Smithsonian National Zoo Conservation Biology Institute Front Royal Virginia USA; ^3^ Department of Reproductive and Behavioral Sciences Saint Louis Zoo St. Louis Missouri USA; ^4^ Department of Physics Utah Valley University Orem Utah USA; ^5^ Association of Zoos and Aquariums Reproductive Management Center Saint Louis Zoo St. Louis Missouri USA; ^6^ Essex County Turtle Back Zoo West Orange New Jersey USA

**Keywords:** adrenal activity, estrus, felidae, *Neofelis nebulosa*, reproductive hormones

## Abstract

Clouded leopards are notoriously difficult to manage under human care due to their tendency toward high stress, intersex aggression, and limited reproductive success. This case study investigated the endocrinological interplay between adrenal and ovarian steroids and describes a positive correlation between concentrations of fecal estrogen and glucocorticoid metabolites in a female clouded leopard. The female was monitored through two sampling periods approximately 16 months apart, and each data set yielded similar results using a simple linear regression model. The finding of a significant positive correlation between the two steroids represents a novel finding in felids and a hitherto unidentified potential interplay between adrenal and ovarian steroid activity. It also adds to our collective understanding of the effects of estrus on female clouded leopards, impacting conservation efforts as we encourage reproduction in this vulnerable species.

## Graphical abstract

Estrogen and glucocorticoid concentrations trended together in a female clouded leopard.

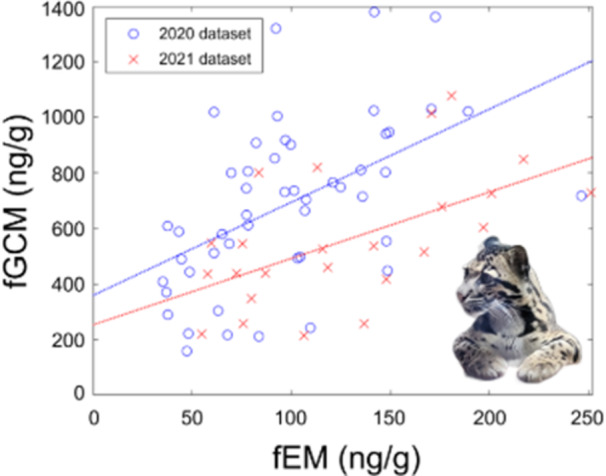

## 1 Introduction

Maintaining ex situ clouded leopard (*Neofelis nebulosa)* populations is vital to conservation of the species. However, there are a number of unique challenges to managing clouded leopards in zoos. Animals are susceptible to environmental stressors and often engage in maladaptive behaviors such as overgrooming or self‐mutilation. They are also difficult to breed, with problems ranging from pair incompatibility and male aggression (Fazio 2010) to unpredictable spontaneous ovulations that limit the success of artificial insemination (Brown 2011).

Fecal hormone analyses are key to studying reproduction and adrenal activity in endangered species, including felids, where 85–95% of steroid metabolites are excreted (Brown 2006). Estrogens play a vital role in the felid ovarian cycle and are highest during estrus, often in association with behavioral signs such as rolling and rubbing and willingness to mate (Fazio 2010). Interestingly, there is a species difference in estrogen pregnancy patterns among cat species, with concentrations increasing after mid‐gestation in cheetahs, Pallas's cats, fishing cats, and domestic cats, but not in clouded leopards or tigers (Brown 2011). Glucocorticoids (GCs) have been used to monitor adrenal function as a proxy for stress in several felid species, including clouded leopards. For instance, in both cheetahs and clouded leopards, individuals whose behavior was described by caretakers as “nervous” had higher fecal GC metabolite (fGCM) concentrations than other individuals (Terio, Citino, and Brown 1999; Wielebnowski 1999). Increases in fGCM concentrations have also been observed during times of illness in fishing cats (Fazio et al. 2020) and anesthesia, translocation, and introduction of a male in cheetahs (Terio, Citino, and Brown 1999). In tigers, fGCM concentrations increased with higher levels of tourism activity in animals living in two wild tiger reserves (Tyagi et al. 2019). Finally, Wielebnowski, Fletchall, et al. (2002) revealed predictable correlations between fGCM concentrations and environmental factors such as exhibit height, time spent with keepers, and visual contact with other predators in zoo‐housed clouded leopards, and that females consistently demonstrated higher concentrations than males.

As such, there exists a longstanding assumption that stress is an underlying factor responsible for the failure to reproduce under human care across felid species, with clouded leopards being particularly affected (Brown 2006, 2011). Monitoring both ovarian and adrenal steroids could shed light on factors affecting reproductive success, so the aim of this technical report was to determine if or how measures of fecal estrogen metabolite (fEM) and fGCM concentrations are correlated in this species.

## 2 Methods

### 2.1 Fecal Sample Collection

Fecal samples were collected from a female clouded leopard at the Loveland Living Planet Aquarium in Draper, Utah. This individual was paired and continually housed with a single male from the age of 8 months old. Despite continuous access to the male and at least one reported observation of copulation in the years before the sampling periods, this female never became pregnant. Fecal samples were collected at least every other day over two time periods 16.5 months apart: 9 weeks (April 1 to June 2) in 2020 and 7 weeks (October 20 to December 1) in 2021. The female was 6 years old during the first sampling period and 7 years old during the second. Copulation was not observed during either trial period, and neither did she become pregnant. Between the two sampling periods, the female received a Suprelorin implant, although the implant was nonfunctional during the second sampling period, as evidenced by a return to estrous cyclicity based on the fEM profile. Feces were collected within 24 h of voiding and stored in plastic bags at –20°C. In 2020, samples were analyzed at the Saint Louis Zoo (SLZ), in St. Louis, Missouri, while the second set was analyzed at the Smithsonian National Zoo and Conservation Biology Institute (SNZCBI) in Front Royal, Virginia. Teal Wilton food dye was added to the female's meat to differentiate her feces from the male's.

Because some samples in 2020 were collected during a pandemic‐related closure of the aquarium to visitors, an analysis was conducted to test for correlations between fEM and fGCM concentrations and visitor numbers.

### 2.2 Fecal Processing and Steroid Analyses

#### 2.2.1 SLZ Methods

Fecal steroids were solubilized using a previously published method (Kozlowski et al. 2020). Briefly, approximately 0.5 g of wet fecal material was shaken overnight in 5 mL modified phosphate‐saline buffer containing 50% methanol. Following centrifugation at 4000*g* for 60 min, supernatants were decanted and stored in evaporation‐proof vials at −80°C until assay. Solid fecal material remaining in the extraction vials was weighed after drying overnight at 80°C. Hormone concentrations were determined as ng/mL, and then divided by the dry weight of the extracted fecal material to give the results as ng/g dried feces.

Fecal estrogen metabolites were quantified by enzyme immunoassay (EIA) (DetectX Estradiol EIA K030, Arbor Assays, Ann Arbor, MI, USA) following a 1:10 dilution in assay buffer. Lower and upper detection limits were 0.039 and 10 ng/mL, respectively. Fecal GCs were quantified using a corticosterone radioimmunoassay (DA I‐125 Corticosterone RIA, ICN MP Biomedicals, Solon, OH, USA). Lower and upper detection limits were 0.26 and 20 ng/mL, respectively. The assay was performed according to the manufacturer's protocol except for the addition of standard diluent to the fecal extracts, and extraction buffer (containing 50% methanol) to the standards.

Intra‐assay variation was 9.2% for estradiol and 8.1% for corticosterone. Inter‐assay coefficients of variation for two quality control pools were 8.8% for estradiol and 9.8% for corticosterone.

### 2.3 SCBI Methods

Fecal processing and steroid analyses followed the protocols of Putman et al. (2015). Feces were dried in a lyophilizer, sifted, and 0.2 g extracted twice by mixing with 5 mL of 90% ethanol on a multi‐tube vortex (Glas‐Col, Terra Haute, IN) for 30 min. Supernatants were combined and evaporated to dryness, resuspended in 1 mL methanol, and dried again; final extracts were re‐suspended in 1 mL assay buffer (X065; Arbor Assays, Ann Arbor, MI) and stored at −20°C until analysis.

Fecal estrogens were analyzed by double‐antibody EIA using a polyclonal primary antibody rabbit anti‐estradiol (R4972, 1:40,000, C. Munro, University of California, Davis, CA), estradiol standards (0.098–25 ng/mL), and estradiol‐HRP (1:110,000, C. Munro).

The double‐antibody EIA for fGCMs utilized a polyclonal antibody (CJM006, 1:60,000, C. Munro), corticosterone standards (0.08–20 ng/mL), and corticosterone‐HRP (1:25,000, Boisseau N, SCBI). Intra‐assay variation was 6.4% for estradiol and 7.2% for corticosterone. Inter‐assay coefficients of variation for two quality control pools were 8.5% for estradiol and 9.2% for corticosterone.

Assays were validated for clouded leopards by demonstrating significant recovery of exogenous steroid added to standards before analysis (> 90%), and parallelism between extract dilutions and the respective standard curves. The biological relevance of the MP Bio corticosterone RIA was demonstrated using an ACTH challenge (Wielebnowski, Fletchall, et al. 2002). The SCBI lab then validated the CJM0006 antibody by cross‐comparing results between the RIA and the EIA, showing excellent agreement (*r* > 0.85) and similarity in longitudinal profiles. Estradiol‐17β is excreted in its native form in clouded leopards (Brown et al. 1994). The original estradiol assay was a tritiated RIA using an antibody against estradiol‐17β‐6‐o‐carboxy‐methyl‐3‐oxime:BSA (Brown et al. 1994). As that antibody ran out, we validated a polyclonal rabbit anti‐estradiol estradiol generated against the same antigen (R4972, C. Munro, University of California, Davis, CA) that correlated highly (*r* > 0.87) that produced similar longitudinal profiles. The Arbor Assays estradiol assay also has been shown to be comparable to the R4972 EIA; that is, highly correlated (*r* > 0.91) with comparable estrous patterns in unpublished data.

### 2.4 Data Analysis

The data were analyzed using a simple linear regression model and plotted with fEM as the independent variable and fGCM as the dependent variable. MATLAB (MathWorks Inc., Natick, MA) was used for regressions and analysis of covariance (ANCOVA) calculations. Significance was assessed by performing a *t* test on the regression slope.

### 2.5 Ethics Statement

Ethical approval was not sought for this technical report because all data was collected non‐invasively and no humans or animals were directly affected by this study.

## 3 Results

**Figure 1:**
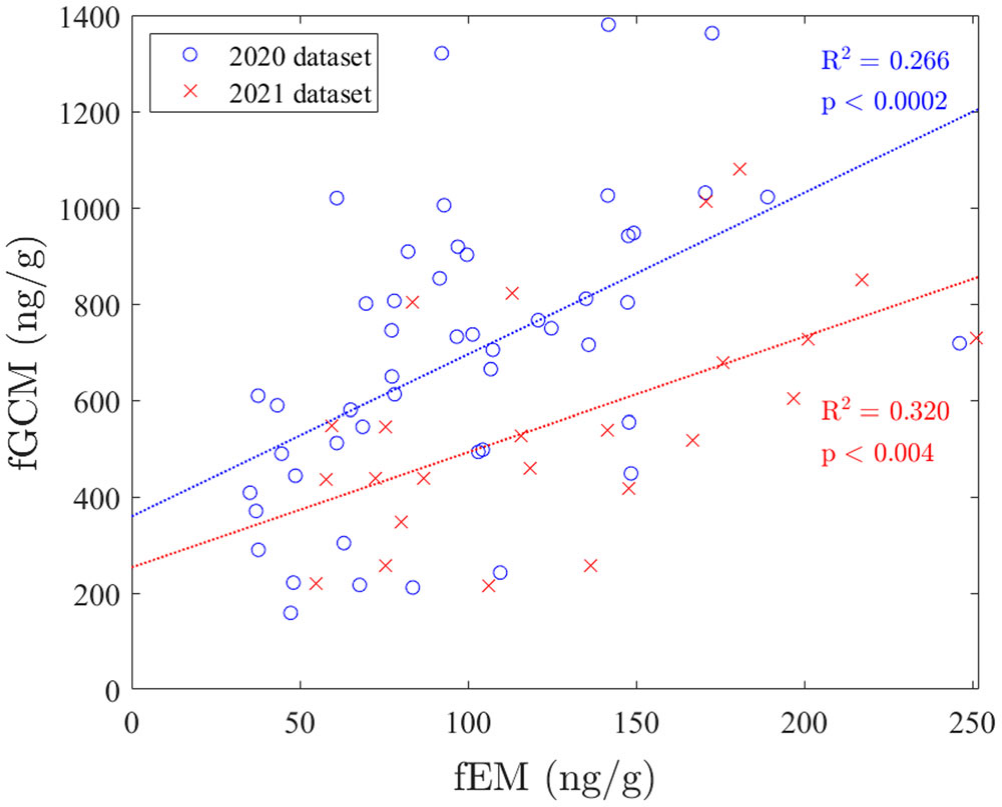
Correlation between fecal glucocorticoid (fGCM) and estrogen (fEM) metabolite concentrations for the 2020 and 2021 data sets.

Linear regressions for both 2020 and 2021 data sets (Figure 1) revealed positive correlations between fEM and fGCM concentrations (2020 data set: fGCM = 3.4 × fEM + 360, *R* ^2^ = 0.27, *p* = 0.000148; 2021 data set: fGCM = 2.4 × fEM + 250, *R* ^2^ = 0.32, *p* = 0.00405). ANCOVA showed no difference between the slopes of regressions across time periods (*p* = 0.413). There were no significant correlations between fEM (fEM = 0.0069 × visitor_count + 96, *R* ^2^ = 0.005, *p* = 0.638) and fGCM (fEM = −0.12 × visitor_count + 730, *R* ^2^ = 0.035, *p* = 0.197) concentrations and visitor numbers.

## 4 Discussion

This study identified a previously unrecognized positive correlation between fEM and fGCM concentrations in a clouded leopard female. If further studies confirm this trend across the species or felids, in general, it suggests adrenal activity is heightened in females during estrus when estrogens are elevated. Because stress can be defined as the biological response elicited when an individual perceives a threat to its homeostasis (Moberg and Mench 2000), it follows that any change has the potential to cause a stress response. One explanation for the relationship observed in this female may be increased attention from the male during estrus. Keepers observed more grooming and chase/play sessions between the male and female during estrus, while the female engaged in more active behaviors like rolling, vocalizing, and lordosis. Furthermore, Erofeeva et al. found that fCGM concentrations in female felids were positively correlated with the number of interactions with males during introductions in Eurasion lynx, Amur leopard cats, bobcats, and domestic cats (Erofeeva et al. 2023).

It is important to note that GCs play an important part in normal metabolic function and are not always indicative of distress. For instance, GC concentrations increased during mating behavior and copulation in wild male tufted capuchin monkeys (Lynch, Ziegler, and Strier 2002) and in response to enrichment in maned wolves (Cummings et al. 2007). Likewise, an increase in GC concentrations was associated with mating in horses (Colborn et al. 1991). This demonstrates that increased cortisol production does not always imply poor welfare (Moberg and Mench 2000). There are also reports of stimuli labeled as “stressors” increasing reproductive activity in mammals such as in bulls and boars (Tilbrook 2000).

Correlations between gonadal steroids and GCs have been noted in other species, but not consistently, in part because although studies often measure both gonadal and adrenal steroids, it is less common to correlate them. Examples of a positive correlation between cortisol and testosterone include male Asian elephants during musth based on blood (Brown et al. 2007) and fecal (Kumar et al. 2014) analyses. Fecal androgens and fGCM also were correlated in male lions (Putman et al. 2019), but not between fEM and fGCM in females (Putman et al. 2015). Relationships between patterns of fEM and fGCM have not been found in margays, tigrinas, ocelots (Moreira et al. 2007) or cheetahs (Wielebnowski, Ziegler, et al. 2002), though it is worth noting that the cats in these studies were not housed with males during the sampling period and the clouded leopard in this report was housed with a male. Thus, assessments of additional clouded leopards are needed to establish if this is characteristic of the species, an individual anomaly, or an effect of housing with a male. The potential relationship between adrenal activity and estrus, however, suggests that keepers should be especially vigilant in identifying and addressing increases in stress‐induced self‐destructive behaviors, such as overgrooming, during estrus. Understanding the physiological effects of estrus on female clouded leopards can aid in maintaining overall health and promote successful reproduction in the species.

## Conflicts of Interest

The authors declare no conflicts of interest.

## Data Availability

The data that support the findings of this study are available from the corresponding author upon reasonable request.
